# Medical student views on returning to clinical placement after months of online learning as a result of the COVID-19 pandemic

**DOI:** 10.1080/10872981.2020.1800981

**Published:** 2020-07-29

**Authors:** Maria M. Hickland, Eleanor R. Gosney, Katie L. Hare

**Affiliations:** Faculty of Life Sciences and Medicine, King’s College London School of Medical Education, London, UK

**Keywords:** COVID-19, medical student, clinical placement, medical education, tomorrow’s doctors

Medical education has faced drastic changes as a result of the COVID-19 pandemic. The suspension of clinical placements across medical schools from March 2020 resulted in online delivery of the medical curricula through the use of virtual platforms. The recent evaluation of online medical education by our colleagues highlighted key advantages to these novel methods of teaching, including increased accessibility and higher levels of engagement [1,2]. However, as senior clinical-year students are now either preparing to, or have, returned to placements within hospital and community settings, the insurance of medical student safety whilst ensuring the preparedness of the future doctors of tomorrow is paramount. As final year medical students returning to placement, we highlight key areas for discussion amongst medical schools and hospital undergraduate administrators amid the COVID-19 pandemic.

The General Medical Council (GMC) states medical students as tomorrow’s doctors, thus it is imperative that clinical-year medical students return to placements to ensure the continued development of core competencies, outlined in the Outcomes for Graduates by the GMC [3]. As final-year medical students due for graduation in 2021, we look forward to this return due to the limitations in patient-contact during online learning, as well as reduced opportunities in developing our communication skills and the challenges in remote non-clinical working.

Our enthused return to placement is accompanied by a widespread feeling of anxiety amongst ourselves and our peers. The British Medical Association recently released a statement highlighting some of the issues and possible solutions regarding returns to clinical placements, but we question as to whether this goes far enough in alleviating student anxieties, particularly those with pre-existing health conditions or at-risk backgrounds such as ethnic minorities [4].

Whilst we anticipate that the skills we have developed during the COVID-19 pandemic through online teaching will ensure a fluent return to placement, we are concerned about widespread lack of confidence in communication and clinical skills upon our return to placement, especially when compared to previous cohorts. Will our colleagues be understanding, or adjust their expectations if our clinical competencies differ from previous final years?

Much like other health care professionals working on the front line during the COVID-19 pandemic, medical student anxiety remains high, with particular concerns surrounding their interactions with non-medical family members and friends. Medical schools have required students to undertake a risk assessment prior to returning to placement, and whilst we have been assured that appropriate personal protective equipment (PPE) will be provided, this does not negate the fear of contracting the virus. There is particular concern amongst individuals returning to placement who live with at-risk family members, thus assurances in how risk of transmission and infection are being managed is vital to help ease student fears. One study found that a third of medical students preferred not to return to the clinical setting during the pandemic, a higher proportion of which was in the lower pre-clinical years. Those not wishing to return also had a statistically significantly higher level of perceived personal risk compared to those who wished to return, highlighting the concern about risk to self [5].

The impact of COVID-19 has been a global challenge, but as medical students we have found ourselves at an impasse – conflicted between the desire to return to a clinical environment, and the safety of online learning, for our own and others health. We hope that with the continuing improvements in safety measures will not only help negate some of these fears, coupled with the support of both medical schools and hospital staff, students will feel well-supported upon placement return and continue to thrive as tomorrow’s doctors.

## References

[1] Sandhu P, de Wolf M. The impact of COVID-19 on the undergraduate medical curriculum. Med Educ Online. 2020;25(1):1764740. 10.1080/10872981.2020.1764740 PMC7269089 32400298

[2] Hammond D, Louca C, Leeves L, et al. Undergraduate medical education and Covid-19: engaged but abstract. Med Educ Online. 2020;25(1):1781379. 10.1080/10872981.2020.1781379 PMC7482786 32543292

[3] General Medical Council. Outcomes for graduates. 2018. GMC publications, London, UK; 2018.

[4] British Medical Association. Position statement: implications of resuming clinical placements during the COVID-19 pandemic [Internet]. 2020 [cited 2020 716]. Available from: https://www.bma.org.uk/media/2857/20200260-implications-of-resuming-clinical-placements-during-the-covid-19-pandemic-1.pdf

[5] Compton S, Sarraf‐Yazdi S, Rustandy F, et al. Medical students’ preference for returning to the clinical setting during the COVID‐19 pandemic. Med Educ. 2020. DOI:10.1111/medu.14268 PMC7300867 32519383

